# Effects of Probiotic Compounds as Feed Additives on Performance, Rumen Fermentation and Metabolic Blood Indices During the Preweaning Period of Holstein Dairy Calves

**DOI:** 10.3390/ani16152286

**Published:** 2026-07-23

**Authors:** Xusheng Hao, Haotian Yu, Guifang Cui, Jiaming Li, Yujun Jiang, Xuelian Feng, Shanshan Ju, Dewei Wang, Feng Gao

**Affiliations:** 1 College of Animal Science, Inner Mongolia Agricultural University, Hohhot 010018, China; 13789514505@163.com (X.H.); yht12126@163.com (H.Y.); lijiaming1013@163.com (J.L.); jyj1597752673@163.com (Y.J.); f1143042763@163.com (X.F.); 18704804340@163.com (S.J.); devwang@yeah.net (D.W.); 2 Inner Mongolia COFCO Feed (Hohhot) Co., Ltd., Hohhot 011500, China; cuiguifang@126.com; 3 Ruminant Healthy Farming Industry Technology Innovation Consortium, Hohhot 011500, China

**Keywords:** dairy calf, early-life nutrition, probiotic consortium, rumen development

## Abstract

Probiotics are promising alternatives to antibiotics in calf rearing, but effects of *Lactobacillus* species combined with *Bacillus subtilis* on preweaning development remain unclear. Two probiotic consortia—*L. plantarum* + *B. subtilis* (TG1) vs. *L. acidophilus* + *B. subtilis* (TG2)—were compared in Holstein calves. Forty-eight newborn calves (initial BW 37.0 ± 2.7 kg) were randomly assigned to control (CG) or probiotic groups (TG1, TG2) from day 3 for 60 days. Growth, fecal scores, serum biochemistry, antioxidant/immune indices, rumen fermentation, 16S rRNA, and untargeted metabolomics were measured. Compared with CG, TG1 increased final body weight (*p* = 0.002) and average daily gain (ADG, *p* = 0.002), whereas TG2 did not differ from CG in either parameter. Both TG1 and TG2 decreased feed conversion ratio (FCR, *p* < 0.001), with TG1 showing a lower value than TG2. At day 60, TG1 had greater body oblique length than TG2 (*p* = 0.030), whereas neither TG1 nor TG2 differed from CG. Serum analysis: TG1 decreased ALT (*p* = 0.039) and increased CAT (*p* = 0.024) and HDL-C (*p* = 0.047) compared with CG, while TG2 did not differ from CG in these parameters. Both TG1 and TG2 decreased CRE compared with CG (*p* < 0.001). TG2 increased GSH-Px compared with CG and TG1 (*p* = 0.044), whereas TG1 did not differ from CG. Rumen fermentation: both TG1 and TG2 increased BCP compared with CG (*p* = 0.009); TG1 elevated isobutyrate compared with CG (*p* = 0.030), whereas TG2 did not differ from CG; TG2 reduced NH_3_-N compared with CG (*p* = 0.026), whereas TG1 did not differ from CG. No α/β-diversity differences were observed, but taxa were modulated: TG1 enriched *Actinobacteria* and *Olsenella*; TG2 enriched *Bacteroidetes*, *Prevotella*, and *Ruminococcus*. Metabolomics: TG1 upregulated steroid hormone and phenylalanine metabolism; TG2 enhanced unsaturated fatty acid biosynthesis. Correlation networks linked *Ruminococcus* and *UCG-005* to antioxidant status and VFA profiles. These findings indicate that different probiotic consortia are associated with distinct physiological outcomes in preweaning calves, supporting consortium selection for targeted feeding.

## Simple Summary

Newborn dairy calves often face health challenges like diarrhea, which can slow their growth and affect their future milk production. Probiotics—beneficial bacteria added to feed—may help, but different types of bacteria might work in different ways. In this study, we tested two probiotic mixtures in 48 newborn calves over 60 days. One mixture contained *Lactobacillus plantarum* plus *Bacillus subtilis*; the other contained *Lactobacillus acidophilus* plus *Bacillus subtilis*. We measured calf growth, health indicators, blood markers, and the bacterial community in their rumen. Calves that received the first mixture grew faster and used feed more efficiently. Calves that received the second mixture also used feed better and showed improved protein utilization and antioxidant activity. Both mixtures changed the types of bacteria in the rumen, but in different ways. These results suggest that choosing the right probiotic mixture could help farmers raise healthier calves, reduce the need for antibiotics, and support more sustainable farming practices. This research provides a scientific basis for designing targeted probiotic strategies in dairy calf rearing.

## 1. Introduction

The preweaning period is critical for dairy calves, yet it is frequently accompanied by diarrhea and respiratory disease, which impair growth and increase culling rates [1]. Neonatal calves possess immature immune and digestive systems, rendering them highly susceptible to enteric pathogens and environmental challenges [2,3]. Indeed, the incidence of diarrhea during the preweaning period can be two to three times higher than in later stages, with long-term consequences for growth and lifetime productivity [4,5].

Probiotics—live microorganisms that confer health benefits when administered in adequate amounts—offer a promising strategy for mitigating these problems [6]. The establishment of a balanced gastrointestinal microbiota in early life is crucial for lifelong health; for example, a higher abundance of *Faecalibacterium prausnitzii* in young calves is associated with reduced diarrhea [7,8], and enrichment of *Lactobacillus* or *Bacillus* in the ileum correlates with upregulation of mucosal immune genes [9]. During the preweaning period, administration of specific probiotics such as *Lactobacillus*, *Saccharomyces cerevisiae*, or *Clostridium butyricum* can inhibit pathogens through competitive exclusion, enhance gut barrier function, and reduce diarrheal incidence [8,10,11].

Among lactobacilli, *Lactobacillus plantarum* and *Lactobacillus acidophilus* are well-recognized for their probiotic properties, including pathogen inhibition and immunomodulation [12,13]. Nevertheless, they possess distinct functional attributes: *L. plantarum* exhibits robust environmental adaptability and broad metabolic capacity, whereas *L. acidophilus* adheres strongly to mucosal surfaces and reinforces gut barrier integrity. *Bacillus subtilis*, commonly used in ruminant feeds, improves rumen fermentation, nutrient digestibility, and reduces diarrhea in calves [14,15] by producing digestive enzymes and enhancing microbial protein synthesis [16].

Despite the known benefits of individual probiotic strains, direct comparisons of how each *Lactobacillus* species, when combined with *B. subtilis*, differentially influences multiple facets of preweaning calf development—including growth, systemic metabolism, rumen fermentation, and the underlying rumen microbial networks—are lacking. Therefore, this study employed a multi-dimensional approach to evaluate the effects of two probiotic consortia (*L. plantarum* + *B. subtilis* vs. *L. acidophilus* + *B. subtilis*) in preweaned dairy calves. The efficacy of each consortium was assessed by measuring growth performance, serum biochemical and antioxidant/immune markers, rumen fermentation parameters, 16S rRNA-based rumen bacterial community, and untargeted rumen metabolomics. By integrating these data, we aimed to establish a holistic understanding of how these two consortia differentially affect calf physiology. Based on the distinct functional traits of the two Lactobacillus species, we hypothesized that the two probiotic consortia would exert divergent physiological effects in preweaning calves: the *L. plantarum* + *B. subtilis* combination may preferentially modulate growth performance and systemic antioxidant status, while the *L. acidophilus* + *B. subtilis* combination may preferentially affect ruminal nitrogen metabolism and microbial protein synthesis. This head-to-head comparison with an identical *B. subtilis* background helps to disentangle the strain-specific contributions of the two Lactobacillus strains. Our findings are expected to guide the rational selection of probiotic consortia for improving growth performance and rumen functional development, thereby contributing to sustainable, antibiotic-free calf rearing systems.

## 2. Materials and Methods

### 2.1. Ethical Statement

All experimental procedures were approved by the Animal Care and Ethics Committee of Inner Mongolia Agricultural University (approval number NND 2024011). Animals were managed in accordance with the institutional guidelines for the care and use of laboratory animals in China.

### 2.2. Experimental Design, Animals, and Management

A total of 48 newborn female Holstein calves with similar birth weight (37.02 ± 2.67 kg) and confirmed good health status were used in a completely randomized design. Health was assessed by the herd veterinarian based on alertness, normal posture and movement, and a strong suckling reflex. Calves were born over a period of approximately 5 days. To control for potential age effects, calves were blocked by birth date and randomly assigned to treatments within each birth cohort. All calves within the same birth cohort were enrolled at 3 days of age. All calves received 4 L of pasteurized colostrum via nipple bottle within 2 h after birth, followed by an additional 2 L at 6 h postpartum. According to the experimental farm’s standard operating procedures, colostrum from each batch was tested using a calibrated Brix refractometer, and only colostrum with a Brix value ≥ 25% was retained for feeding and pasteurized before administration.

Calves were randomly allocated to one of three dietary treatments (*n* = 16 per group): a control group (CG) fed a basal starter diet; test group 1 (TG1) fed the basal diet supplemented with *Lactobacillus plantarum* and *Bacillus subtilis*; and test group 2 (TG2) fed the basal diet supplemented with *Lactobacillus acidophilus* and *Bacillus subtilis*. The compound probiotic product (Beijing Biotechnology Co., Ltd., Beijing, China) contained *L. plantarum* CGMCC No. 12554, *L. acidophilus* CGMCC No. 1.12735, and *B. subtilis* CGMCC No. 27341—all deposited in the China General Microbiological Culture Collection Center (CGMCC), a nationally recognized culture collection. These strains are approved as safe microbial feed additives for ruminants in China per the Catalog of Feed Additive Varieties (2023 Edition) (Ministry of Agriculture and Rural Affairs, China), and have completed standardized safety evaluations covering pathogenicity, virulence factor screening, and acquired antibiotic resistance gene detection. Probiotics were uniformly incorporated into the pelleted starter at an inclusion rate of 500 g per metric ton (0.05%). This dosage was determined based on the manufacturer’s standard recommendation for calf starters. The viability and counts of the probiotic strains were assured by the manufacturer’s quality control system. The probiotics were produced using microencapsulation technology to enhance stability during feed processing and storage. Probiotic supplementation began at 3 d of age and continued throughout the 60 d trial. The composition and nutrient levels of the basal diet, which was formulated according to the NRC (2001) [17] nutrient requirements for Holstein calves, are presented in Table 1.

From 3 d of age, calves were housed individually in hutches (1.2 m × 2.4 m) with adjacent exercise pens (1.2 m × 1.5 m), following the “one calf, one hutch” principle. The facility was kept clean, dry, and well-ventilated. From summer, calves had ad libitum access to water at ambient temperature. Bedding was maintained at a minimum thickness of 20 cm and kept loose and dry. Hutches were cleaned and disinfected daily; manure was removed before disinfection. Feed and water buckets were washed daily after the morning feeding. Whole milk was fed twice daily (08:00 and 16:30) after pasteurization and warming to 39–40 °C. Milk feeding volumes followed a standardized step-down schedule: 5–6 L/d from d 1 to 3, 5–7 L/d from d 4 to 7, 7–8 L/d from d 8 to 15, 8–10 L/d from d 16 to 30, and 10–12 L/d from d 31 to 60.

### 2.3. Sample Collection and Measurement Methods

#### 2.3.1. Starter Intake and Growth Performance

Calves were weighed and body measurements (withers height, body length from shoulder to pin bone, and heart girth behind the shoulders) were recorded on d 1, 30, and 60 before the morning feeding. Daily feed intake and orts were recorded. Average daily gain (ADG), DM intake (DMI), and feed conversion ratio (FCR; DMI/ADG) were calculated.

#### 2.3.2. Blood Sample Collection and Biochemical Analyses

On day 60, before the morning feeding, venous blood samples (10 mL) were collected from 10 calves per group from the jugular vein using sterile disposable needles, and no baseline samples were taken prior to treatment allocation. Serum was separated through centrifugation at 1200× *g* for 10 min and stored at –20 °C until analysis.

Concentrations of tumor necrosis factor-α (TNF-α), interferon-γ (IFN-γ), interleukin-2 (IL-2), IL-6, IL-10, immunoglobulin G (IgG), IgM, and IgA were measured using commercial ELISA kits according to the manufacturers’ instructions. Antioxidant parameters, including total antioxidant capacity (T-AOC), superoxide dismutase (SOD), glutathione peroxidase (GSH-Px), catalase (CAT), and malondialdehyde (MDA), were assessed using kits from Nanjing Jiancheng Biotechnology Co., Ltd., Nanjing, China.

Biochemical markers—aspartate aminotransferase (AST), alanine aminotransferase (ALT), alkaline phosphatase (ALP), albumin (ALB), triglycerides (TG), total proteins (TP), glucose (GLU), cholesterol (CHOL), high-density lipoprotein cholesterol (HDL-C), low-density lipoprotein cholesterol (LDL-C), and creatinine (CRE)—were analyzed using an automated biochemical analyzer (Hitachi 3100, Hitachi High-Tech Corporation, Tokyo, Japan).

#### 2.3.3. Rumen Fluid Collection and Fermentation Analysis

On day 60 of the formal feeding trial, 10 calves were randomly selected from each group two hours after the morning feeding, and approximately 100 mL of rumen fluid was collected from each calf using a Kelibo bovine stomach tube (Kelibo Equipment Co., Ltd., Wuhan, China). The first 50 mL was discarded to avoid salivary contamination. The remaining fluid was filtered through four layers of sterile medical gauze and aliquoted into 15 mL centrifuge tubes. The pH was measured immediately using a calibrated pH meter (PHS-3C, Shanghai LeiCi Device Works, Shanghai, China). The fluid was then centrifuged at 2800× *g* for 15 min at 25 °C to obtain supernatant [18].

For ammonia nitrogen (NH_3_-N) determination, 0.5 mL of supernatant was mixed with 4.5 mL of 0.2 mol/L HCl. For volatile fatty acid (VFA) analysis, 4 mL of supernatant was mixed with 1 mL of 25% metaphosphoric acid. Bacterial crude protein (BCP) was determined from the pellet using differential centrifugation, ultrasonication, and Coomassie Brilliant Blue staining, with absorbance measured at 595 nm [19]. NH_3_-N was measured colorimetrically, and VFA concentrations were analyzed by gas chromatography (GC2010, Shimadzu Corporation, Kyoto, Japan).

#### 2.3.4. Determination of Rumen Microbial Community

Total microbial DNA was extracted from rumen samples using the E.Z.N.A.^®^ Soil DNA Kit (Omega Bio-tek, Norcross, GA, USA) following the manufacturer’s protocol. DNA integrity was verified through 1% agarose gel electrophoresis, and concentration and purity were assessed with a NanoDrop2000 spectrophotometer (Thermo Scientific, Waltham, MA, USA). The V3–V4 hypervariable region of the 16S rRNA gene was amplified using primers 338F (5′-ACTCCTACGGGAGGCAGCAG-3′) and 806R (5′-GGACTACHVGGGTWTCTAAT-3′). PCR was performed in 20-μL reactions containing 4 μL 5× FastPfu buffer, 2 μL 2.5 mM dNTPs, 0.8 μL each forward/reverse primer (5 μM), 0.4 μL FastPfu polymerase, 0.2 μL BSA, and 10 ng template DNA. Thermal cycling conditions were: 95 °C for 3 min; 27 cycles of 95 °C for 30 s, 60 °C for 30 s, 72 °C for 30 s; and a final extension at 72 °C for 10 min. PCR products were purified using magnetic beads and quantified with a Qubit 4.0 fluorometer (Thermo Fisher, Waltham, MA, USA). Equal amounts of purified amplicons were pooled for sequencing on an Illumina MiSeq platform (Illumina, Inc., San Diego, CA, USA).

Raw sequences were quality-filtered, merged [20], and clustered into operational taxonomic units (OTU) at 97% similarity using UPARSE v7.1 [21]. Chimeric sequences were removed. To normalize sequencing depth, all samples were rarefied to 6000 sequences, achieving an average Good’s coverage of 99.09%. A rarefaction depth of 5000–10,000 sequences per sample is commonly used for comparing bacterial communities in rumen and other gut environments [22,23]. Taxonomic assignment was performed with the RDP classifier (v2.11) against the Silva 16S rRNA database (v138) at a 70% confidence threshold [24]. Alpha diversity indices (Chao1, Shannon) were calculated, and beta diversity was visualized through principal coordinate analysis (PCoA) based on Bray–Curtis distances. Permutational multivariate analysis of variance (PERMANOVA) was used to test for differences in microbial community structure. Functional profiles were predicted using PICRUSt2 v2.2.0. All analyses were performed on the Majorbio Cloud Platform (https://cloud.majorbio.com).

#### 2.3.5. Metabolomics Analysis and Data Processing

Rumen fluid samples were analyzed through ultra-performance liquid chromatography tandem mass spectrometry (UPLC-MS/MS) using a Waters Acquity I-Class PLUS UHPLC system coupled to a Xevo G2-XS QTOF high-resolution mass spectrometer (Waters Corporation, Milford, MA, USA). Chromatographic separation was achieved on a Waters Acquity UPLC HSS T3 column (2.1 × 100 mm, 1.8 μm) maintained at 40 °C. The mobile phase consisted of (A) 0.1% formic acid in water and (B) 0.1% formic acid in acetonitrile. A 2-μL injection volume was used, and the flow rate was 0.4 mL/min. Mass spectra were acquired in both positive and negative electrospray ionization modes over an *m*/*z* range of 50–1200. Raw data were processed using Progenesis QI software v3.0 for peak picking, alignment, and compound identification against the METLIN database and an in-house spectral library. Metabolites were annotated using the KEGG, HMDB, and LipidMaps databases. Differential metabolites were identified based on fold change (FC ≥ 2 or ≤0.5), *p* < 0.05 (Student’s *t*-test), and variable importance in projection (VIP) > 1 from orthogonal partial least squares discriminant analysis (OPLS-DA). Pathway enrichment analysis was performed using KEGG pathways with hypergeometric testing.

### 2.4. Statistical Analysis

Preliminary data processing was conducted utilizing Excel (Microsoft) software. All statistical analyses were performed using SAS 9.2 (SAS Institute Inc.). Repeated Measurement Indicators (body size) were analyzed using the PROC MIXED procedure (mixed model). The statistical model was defined as:

Yijk = μ + τi + βj + τβij + Ck (i)+ εijk

where Yijk = the k-th observation of the dependent variable in the i-th treatment group and j-th time point; μ = overall mean; τi = fixed effect of compound probiotics level (i = 1, 2, 3, corresponding to control, TG1, TG2 groups); βj = fixed effect of time point (j = 1, 2, 3, corresponding to 1 d, 30 d, 60 d); τβij = fixed interaction effect of treatment and time; Ck (i) = random effect of the kth calf within the i-th treatment group; and εijk = random error.

Independent Indicators (body weight, DMI, ADG, FCR, blood indices, rumen fermentation parameters, α-diversity) were analyzed using the PROC GLM procedure (one-way ANOVA). Multiple comparisons were performed using Tukey’s test. Final results were presented as the mean and standard error of the mean (SEM). A *p*-value < 0.05 was significant, and a *p*-value < 0.01 was extremely significant.

## 3. Results

### 3.1. Growth Performance

Table 2 presents the growth performance of calves fed diets supplemented with different probiotic formulations. No significant differences were observed in initial body weight among the groups. At the end of the 60-day trial, final body weight was significantly higher in the TG1 group compared to the CG (*p* = 0.002), while the TG2 group did not differ significantly from either group. The TG1 group exhibited a significantly higher ADG over the 60-day period than both the CG and TG2 groups (*p* = 0.002). FCR was significantly decreased in both probiotic-treated groups (*p* < 0.001), with the TG1 group demonstrating the highest efficiency.

The effects of the compound probiotic on calf body measurements are shown in Table 3. Across all traits, time exerted a significant main effect (*p* < 0.001). Body height did not differ among groups at any stage (*p* > 0.05), and the probiotic × time interaction was negligible (*p* = 0.973). Likewise, body length revealed no interaction (*p* = 0.491); although initial values were similar, TG1 tended to be longer than TG2 by day 60 (*p* = 0.030). A significant probiotic × time interaction was detected for heart girth (*p* = 0.039), yet mean girth never differed among treatments throughout the trial (*p* > 0.05).

### 3.2. Biochemical Blood Indices Analysis

The results of the effect of compound probiotics on the blood indices of calves are shown in Table 4, Table 5 and Table 6. The ALT in the TG1 group was significantly lower than that of the CG (*p* = 0.039), and CRE in both treatment groups was significantly lower than that of the CG (*p* < 0.001). HDL-C was significantly higher in the TG1 group than in the CG and the TG2 group (*p* = 0.047), and other biochemical markers did not differ significantly between groups (*p* > 0.05) (Table 4). CAT activity was significantly higher in the TG1 group than in the CG (*p* = 0.024), TG2 group GSH-Px activity was significantly higher than the other two groups (*p* = 0.044), and no significant variations were detected in other antioxidant parameters (*p* > 0.05) (Table 5). The level of IL-6 was significantly lower in the TG1 group than in the CG and the TG2 group (*p* = 0.009), and the other immune indexes did not show any differences between groups (*p* > 0.05) (Table 6).

### 3.3. Rumen Fermentation Parameters

The effects of compound probiotics on rumen fermentation parameters are shown in Table 7. Compared to the CG, the TG2 group demonstrated a significant reduction in ruminal NH_3_-N concentration (*p* = 0.026) along with a marked elevation in BCP levels (*p* = 0.009), while maintaining comparable isobutyric acid concentrations. Furthermore, TG1 calves exhibited significantly elevated ruminal concentrations of both BCP and isobutyric acid relative to the CG (*p* < 0.05), with valeric acid levels showing a significant increase compared to TG2 counterparts (*p* = 0.045), and the other fundamental fermentation parameters did not show any differences between groups (*p* > 0.05).

### 3.4. Rumen Microbial Profile

**Figure 1:**
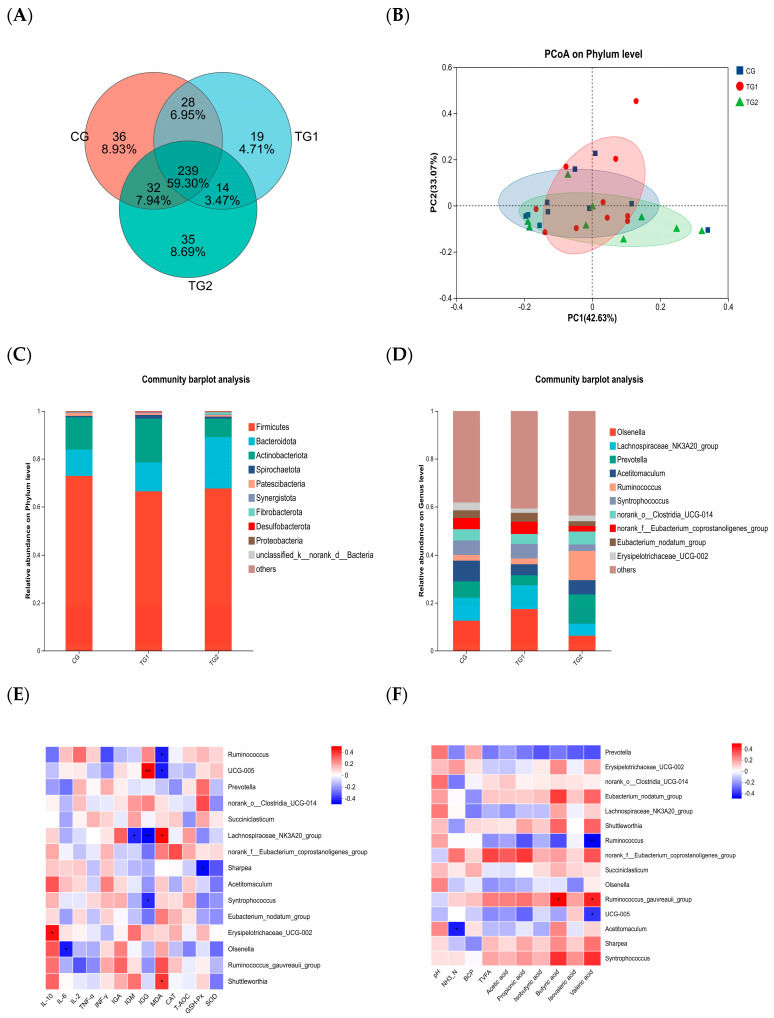
Effect of compound probiotics on the rumen microbial composition and communities of Holstein calves (*n* = 10 per group). 0.01 < *p* ≤ 0.05 *, 0.001 < *p* ≤ 0.01 ** (**A**) Characteristic Venn diagram. (**B**) PCoA analysis of β diversity. (**C**) Distribution of main rumen bacteria at the phylum level. (**D**) Distribution of main rumen bacteria at the genus level. (**E**) Microbial, serum immunity and antioxidant indexes, correlation analysis heatmap. (**F**) Microbial rumen fermentation parameters, correlation analysis heatmap.

Following preliminary quality control and cluster analysis, 335, 300, and 320 OTUs were identified in the CG, TG1, and TG2 groups respectively, with 239 OTUs demonstrating cross-group conservation (Figure 1A). The α-diversity of ruminal microbiota was evaluated through ACE, Chao, Simpson, and Shannon indices (Table 8), while β-diversity reflecting microbial compositional similarity was analyzed via PCoA (Figure 1B). No statistically significant differences (*p* > 0.05) were observed in either α- or β-diversity metrics between the treatment groups (TG1 and TG2) and the control group.

Taxon-dependent analysis revealed the top 10 most abundant phyla and genera across the three groups. At the phylum level, *Firmicutes* dominated the microbial community (mean abundance: 69.01%), followed by *Bacteroidota* (14.67%) and *Actinobacteriota* (3.84%) (Figure 1C). Notably, the TG1 group exhibited elevated *Actinobacteriota* relative abundance (18.18%) compared to CG (13.32%) and TG2 (7.67%), whereas *Bacteroidota* predominance was observed in TG2 (21.40%) relative to CG (11.11%) and TG1 (12.18%).

Genus-level analysis identified *Olsenella* as the predominant taxon in CG (12.50%) and TG1 (17.36%), while *Prevotella* dominated TG2 (12.31%) compared to CG (6.78%) and TG1 (4.16%) (Figure 1D). *Ruminococcus* abundance was substantially lower in CG and TG1 (2.40% each) than in TG2 (12.20%).

Interaction heatmaps elucidated genus-level associations with serum biomarkers (Figure 1E): *Erysipelotrichaceae_UCG-002* exhibited a significant positive correlation with serum IL-10. *Olsenella* demonstrated an inverse correlation with IL-6. *Ruminococcus* and *UCG-005* abundance negatively correlated with MDA levels. *Lachnospiraceae_NK3A20_group* showed dual associations: positive with MDA yet negative with IgM and IgG. *UCG-005* positively influenced IgG concentrations. *Sharpea* displayed complex interactions with GSH-Px.

Further analysis revealed rumen fermentation dynamics (Figure 1F): *Ruminococcus* and *UCG-005* abundance inversely correlated with valerate concentrations. *Ruminococcus_gauvreauii_group* positively associated with butyrate and valerate levels. *Acetitomaculum* abundance showed a negative correlation with NH_3_-N concentrations.

### 3.5. Rumen Microbial Metabolism

**Figure 2:**
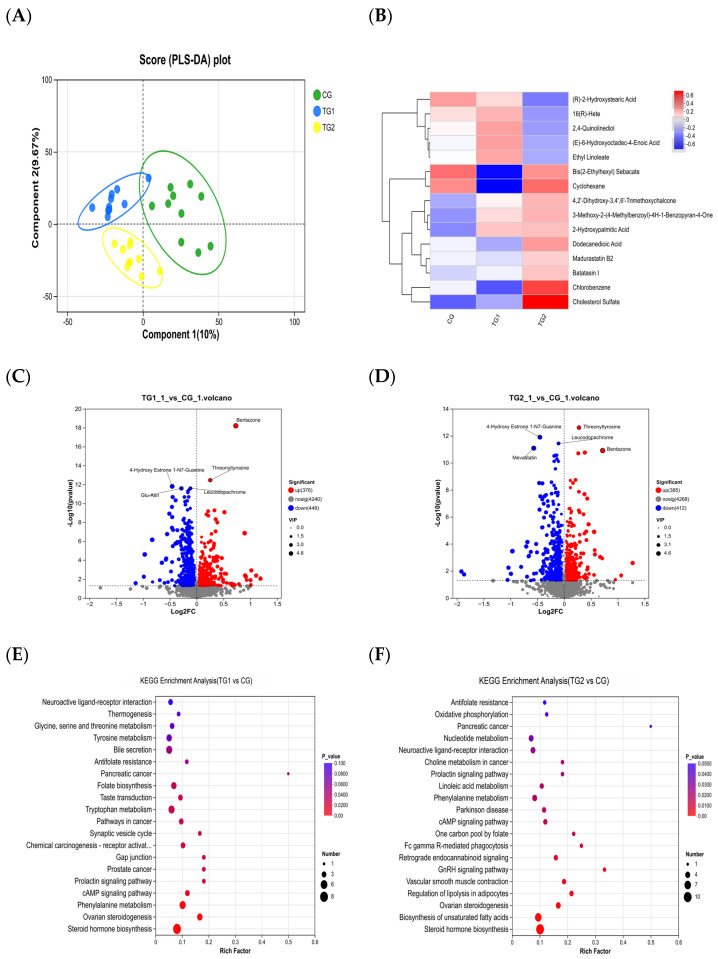
Effect of compound probiotics on the rumen metabolite profiles of Holstein calves (*n* = 10 per group). (**A**) Partial least squares discriminant analysis. (**B**) Metabolite cluster analysis. (**C**,**D**) Differential metabolite volcanic diagram. (**E**,**F**) Differential metabolite KEGG enrichment map.

Partial least squares discriminant analysis (PLS-DA) showed clear separation among the three groups (Figure 2A). A heatmap of the top 15 differential metabolites displayed distinct clustering patterns among CG, TG1, and TG2 (Figure 2B). In TG1, the levels of bis (2-ethylhexyl) sebacate and cyclohexane were lower than those in CG and TG2. In TG2, the levels of chlorobenzene and cholesterol sulfate were higher than in CG and TG1.

Differential metabolites were identified using orthogonal partial least squares discriminant analysis (OPLS-DA) with variable importance in projection (VIP) scores, fold change, and *p*-values, visualized as volcano plots (Figure 2C,D). Compared with CG, TG1 had 240 upregulated and 242 downregulated metabolites; TG2 had 242 upregulated and 236 downregulated metabolites. Compared with TG2, TG1 had 197 upregulated and 124 downregulated metabolites.

KEGG pathway enrichment analysis showed that, relative to CG, TG1 enriched pathways including steroid hormone biosynthesis, ovarian steroidogenesis, and phenylalanine metabolism (Figure 2E). TG2 enriched pathways including biosynthesis of unsaturated fatty acids and regulation of lipolysis in adipocytes (Figure 2F).

**Figure 3:**
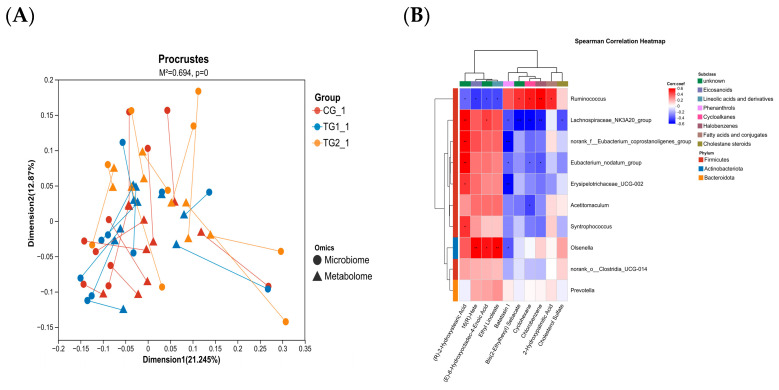
Microbial community structure and metabolic profiles (*n* = 10 per group). 0.01 < *p* ≤ 0.05 *, 0.001 < *p* ≤ 0.01 **, *p* ≤ 0.001 *** (**A**) Procrustes analysis. (**B**) Heatmap of metabolites and interspecies correlations.

Procrustes analysis indicated concordance between microbial community structure and metabolic profiles across groups (Figure 3A). Correlation network analysis (Figure 3B) revealed positive correlations between *Olsenella* abundance and three metabolites: 16 (R)-HETE, (E)-6-hydroxyoctadec-4-enoic acid, and ethyl linoleate.

## 4. Discussion

This study compared two probiotic consortia—*L. plantarum* + *B. subtilis* (TG1) and *L. acidophilus* + *B. subtilis* (TG2)—in preweaning calves. The neonatal calf’s immature physiology creates a window of susceptibility [25], and probiotics may accelerate gut maturation by promoting epithelial proliferation, enhancing barrier function, and establishing a beneficial microbial ecosystem [26,27]. However, the specific effects of different consortia on growth and metabolism remain poorly understood.

Contrary to findings by Wang et al. [28], the growth-promoting effect of TG1 was observed. This effect, along with the decreased FCR in both consortia, suggests that enhanced nutrient utilization may contribute to the performance benefits. The discrepancy between our study and Wang et al. [28] may be attributed to differences in intervention timing (starting at day 3 vs. day 7 of age) and the specific probiotic strains used, as probiotic outcomes are highly context-dependent [29]. The improved body measurements (body oblique length, heart girth) observed in TG1 are consistent with growth-promoting effects reported for *Lactobacillus* strains [30]. In contrast, TG2 did not show improvements in body measurements relative to CG, suggesting that the L. acidophilus-based consortium may have had a more limited impact on skeletal growth under the conditions of this study.

Probiotic supplementation was associated with lower serum creatinine in both TG1 and TG2 and lower ALT in TG1. These changes have been linked to reduced metabolic stress in some studies [31], but their functional significance in the present study remains to be determined. HDL-C was higher in TG1, a change that could reflect altered lipid transport. Consortium-associated differences in antioxidant enzyme activities were observed: TG1 showed higher catalase activity, whereas TG2 showed higher glutathione peroxidase activity. Therefore, the antioxidant effects were enzyme-specific and did not translate into a broad enhancement of systemic antioxidant capacity, as evidenced by the lack of changes in SOD, MDA, and T-AOC.

The significant reduction in the pro-inflammatory cytokine IL-6 in TG1 was observed, but other immune parameters (e.g., IL-10, TNF-α, IgA, IgM, IgG) did not differ significantly between groups. Thus, the anti-inflammatory effect appears limited to IL-6 rather than representing a systemic response. This single change may still be of interest, as IL-6 has been linked to inflammation-associated growth depression in some studies [32,33]. Whether this isolated reduction in IL-6 contributed to improved health or growth warrants further investigation.

Both probiotic groups had higher ruminal BCP compared to CG, suggesting increased microbial protein synthesis. TG2 showed lower ammonia-N concentration, which may indicate more efficient incorporation of ammonia into microbial protein, consistent with studies on Bacillus species [34,35]. TG1 exhibited higher isobutyrate and valerate concentrations, which may reflect alterations in carbohydrate fermentation [36]. However, no corresponding changes were observed in major volatile fatty acids, including propionate. Further investigation is needed to clarify the biological significance of these changes in isobutyrate and valerate for host metabolism.

Despite no changes in overall α- or β-diversity, probiotic supplementation was associated with selective enrichment of specific taxa: TG1 increased *Actinobacteria* and *Olsenella*, while TG2 favored *Bacteroidetes*, *Prevotella*, and *Ruminococcus*. Such selective modulation without altering core diversity has been reported in young animals where age and diet are major drivers [37,38]. *Olsenella* (an acetate producer) showed a negative correlation with IL-6 and positive correlations with certain metabolites (e.g., 16 (R)-Hete, ethyl linoleate). *Ruminococcus* and *UCG-005* were negatively associated with MDA and positively associated with some VFA profiles. These correlations provide testable hypotheses for future mechanistic studies.

Metabolomic analysis provided mechanistic clues: TG1 upregulated steroid hormone biosynthesis and phenylalanine metabolism, which may be linked to the enrichment of *Olsenella* and its production of acetate-a precursor for cholesterol and downstream steroid hormones [39]. Enhanced phenylalanine metabolism has been associated with improved nitrogen retention and protein utilization [40], providing a potential link to the growth differences observed in TG1. In contrast, TG2 upregulated unsaturated fatty acid biosynthesis and adipocyte lipolysis pathways, aligning with the dominance of *Prevotella*-a genus known for its role in lipid metabolism [41]. Regarding antioxidant responses, TG1 showed higher CAT activity, while TG2 showed higher GSH-Px activity, suggesting consortium-associated antioxidant modulation. Other measured antioxidant parameters (SOD, MDA, T-AOC) did not differ significantly between groups, indicating that the antioxidant effects were enzyme-specific rather than global. The enrichment of unsaturated fatty acid biosynthesis in TG2 could be related to the increase in GSH-Px activity, as polyunsaturated fatty acids have been reported to influence selenoenzyme expression [42]. Similarly, the altered energy and protein metabolism in TG1 may be associated with reduced oxidative by-product generation, consistent with the increase in CAT activity. These metabolic shifts provide a plausible basis for the observed physiological outcomes, although further studies are needed to establish whether these associations represent causal links.

Several limitations should be considered when interpreting these results. First, the trial was conducted on a single commercial dairy farm, which limits the generalizability of the findings to other management systems, breeds, or environmental conditions. Second, although the probiotic product was incorporated into the pelleted starter at a defined inclusion rate, individual probiotic CFU intake was not analytically verified; therefore, potential variations in actual intake due to feed processing, storage, or differences in individual starter consumption cannot be excluded. Third, the sample size (*n* = 10–16 per group) was relatively small, particularly for blood and rumen variables where only 10 calves per group were sampled, which reduces statistical power to detect subtle differences between treatments. Fourth, the 60-day preweaning duration does not allow evaluation of long-term effects on post-weaning growth, health, or rumen function. Future multi-farm trials with larger sample sizes, analytically confirmed probiotic intake, and extended post-weaning follow-up are warranted to validate and extend these findings.

## 5. Conclusions

In summary, supplementation with *L. plantarum* + *B. subtilis* was associated with modestly greater body weight gain and alterations in energy and protein metabolism, whereas *L. acidophilus* + *B. subtilis* was associated with lower ruminal ammonia-N concentration and higher microbial protein, along with a specific increase in glutathione peroxidase activity, which may be linked to changes in lipid metabolism. However, the differences observed between the two probiotic consortia were generally modest and not consistent across all measured outcomes. These findings highlight the importance of strain selection and suggest the potential for targeted probiotic strategies during the preweaning period.

## Figures and Tables

**Table 1:** Basic feed composition and nutrient level.

	Diet		
Item	CG	TG1	TG2
Corn	37.00	37.00	37.00
Soybean meal	22.00	22.00	22.00
Cotton meal	11.00	11.00	11.00
DDGS ^1^	7.50	7.50	7.50
Corn husk	3.00	3.00	3.00
Corn germ meal	7.00	7.00	7.00
Wheat bran	5.00	4.95	4.95
Stone meal	1.50	1.50	1.50
Calcium phosphate	0.50	0.50	0.50
Sodium chloride	0.50	0.50	0.50
Premix ^2^	5.00	5.00	5.00
Compound probiotics	-	0.05	0.05
Nutrient composition, %			
DE ^3^, MJ/kg	13.02	13.02	13.02
Dry matter	88.55	88.35	88.37
Ash	6.74	6.74	6.70
Crude protein	22.84	22.84	22.84
Crude fat	2.69	2.67	2.67
Neutral detergent fiber	23.75	23.75	23.75
Acid detergent fiber	17.39	17.39	17.39
Calcium	1.23	1.23	1.23
Phosphorus	0.59	0.59	0.59

Calves were fed whole milk according to the schedule described and had ad libitum access to the basal starter diet shown in Table 1. ^1^ DDGS: Distillers Dried Grains with Solubles. ^2^ Premix provides: vitamin A 18,000 IU; vitamin D_3_ 4000 IU; vitamin E 60 IU; vitamin K_3_ 2.5 mg; vitamin B_1_ 4 mg; vitamin B_2_ 6 mg; niacin 40 mg; pantothenic acid 18 mg; vitamin B_12_ 0.08 mg; Fe 100 mg; Cu 12 mg; Mn 50 mg; Zn 70 mg; Se 0.4 mg; I 0.8 mg; Co 0.15 mg; Ca 4 g; P 3 g; NaCl 2 g; carrier (wheat bran) per kilogram of ration. ^3^ DE: digestive energy.

**Table 2:** Effects of compound probiotics on growth performance of Holstein calves.

	Diet ^1^				
Item	CG (*n* = 16)	TG1 (*n* = 16)	TG2 (*n* = 16)	SEM	*p*-Value
Initial body weight, kg	36.96	36.93	37.04	0.81	0.995
Final body weight, kg	85.61 ^b^	93.96 ^a^	88.61 ^ab^	1.53	0.002
DMI ^2^, kg	1.39	1.39	1.40	0.01	0.422
ADG ^3^, kg/d	0.81 ^b^	0.95 ^a^	0.86 ^b^	0.02	0.002
FCR ^4^	1.72 ^a^	1.46 ^b^	1.63 ^b^	0.01	<0.001

^a,b^ Within a row, means without a common superscript differ (*p* < 0.05). ^1^ Diets were supplemented with different types of probiotic complexes: CG = control without probiotic complex; TG1 = basal diet + *Lactobacillus plantarum* + *Bacillus subtilis*; TG2 = basal diet + *Lactobacillus acidophilus* + *Bacillus subtilis*. Probiotic supplementation began at 3 days of age. ^2^ DMI = total DM of feed, including whole milk and calf starter feed. ^3^ ADG = average daily gain. ^4^ FCR = feed conversion ratio, the ratio of DMI to ADG.

**Table 3:** Effects of compound probiotics on body size of Holstein calves.

	Diet ^1^				
Item	CG (*n* = 16)	TG1 (*n* = 16)	TG2 (*n* = 16)	SEM	*p*-Value
Body height ^2^, cm					
1 d	75.30	75.90	74.18	0.85	0.344
30 d	85.30	85.90	83.64	1.07	0.297
60 d	94.90	94.90	93.27	0.85	0.284
Body oblique length ^3^, cm					
1 d	64.30	67.10	63.09	1.60	0.200
30 d	76.80	79.50	77.45	0.83	0.072
60 d	92.40 ^ab^	93.50 ^a^	90.09 ^b^	0.85	0.030
Heart girth ^4^, cm					
1 d	83.70	83.10	82.73	0.72	0.618
30 d	98.50	97.10	95.18	0.98	0.065
60 d	106.80	109.20	107.82	0.88	0.172

^a,b^ Within a row, means without a common superscript differ (*p* < 0.05). ^1^ Diets were supplemented with different types of probiotic complexes: CG = control without probiotic complex; TG1 = basal diet + *Lactobacillus plantarum* + *Bacillus subtilis*; TG2 = basal diet + *Lactobacillus acidophilus* + *Bacillus subtilis*. Probiotic supplementation began at 3 days of age. ^2^ Body height: overall time effect *p*-value < 0.001; diet × time interaction effect *p*-value = 0.973. ^3^ Body oblique length: overall time effect *p*-value < 0.001; diet × time interaction effect *p*-value = 0.491. ^4^ Heart girth: overall time effect *p*-value < 0.001; diet × time interaction effect *p*-value = 0.039.

**Table 4:** Effects of compound probiotics on serum biochemical indices of Holstein calves.

	Diet ^1^				
Item ^2^	CG (*n* = 10)	TG1 (*n* = 10)	TG2 (*n* = 10)	SEM	*p*-Value
MDA (nmol/mL)	1.87	1.94	1.92	0.08	0.828
SOD (U/mL)	19.59	18.90	19.56	1.02	0.875
T-AOC (mmol/mL)	0.59	0.54	0.58	0.02	0.178
CAT (U/mL)	1.58 ^b^	2.52 ^a^	1.94 ^ab^	0.22	0.024
GSH-Px (U/mL)	123.03 ^b^	126.34 ^b^	155.59 ^a^	9.40	0.044

^a,b^ Within a row, means without a common superscript differ (*p* < 0.05). ^1^ Diets were supplemented with different types of probiotic complexes: CG = control without probiotic complex; TG1 = basal diet + *Lactobacillus plantarum* + *Bacillus subtilis*; TG2 = basal diet + *Lactobacillus acidophilus* + *Bacillus subtilis*. Probiotic supplementation began at 3 days of age. ^2^ MDA = malondialdehyde; SOD = superoxide dismutase; T-AOC = total antioxidant capacity; CAT = catalase; GSH-Px = glutathione peroxidase.

**Table 5:** Effects of compound probiotics on serum antioxidant indexes of Holstein calves.

	Diet ^1^				
Item ^2^	CG (*n* = 10)	TG1 (*n* = 10)	TG2 (*n* = 10)	SEM	*p*-Value
AST (U/L)	47.94	40.54	48.08	3.45	0.227
ALT (U/L)	7.60 ^a^	6.17 ^b^	6.90 ^ab^	0.46	0.039
ALP (U/L)	193.37	192.89	181.19	14.97	0.816
ALB (g/L)	35.36	33.38	34.03	0.78	0.211
CHOL (mmol/L)	2.63	3.07	2.82	0.22	0.386
TG (mmol/L)	0.31	0.42	0.40	0.05	0.293
TP (g/L)	61.57	58.73	59.48	1.28	0.280
CRE (μmol/L)	139.64 ^a^	112.30 ^b^	104.17 ^b^	5.55	<0.001
GLU (mmol/L)	5.33	5.03	5.96	0.47	0.374
HDL-C (mmol/L)	2.50 ^b^	2.98 ^a^	2.44 ^b^	0.16	0.047
LDL-C (mmol/L)	3.44	3.72	3.03	0.27	0.236

^a,b^ Within a row, means without a common superscript differ (*p* < 0.05). ^1^ Diets were supplemented with different types of probiotic complexes: CG = control without probiotic complex; TG1 = basal diet + *Lactobacillus plantarum* + *Bacillus subtilis*; TG2 = basal diet + *Lactobacillus acidophilus* + *Bacillus subtilis*. Probiotic supplementation began at 3 days of age. ^2^ AST = aspartate aminotransferase; ALT = alanine aminotransferase; ALP = alkaline phosphatase; ALB = albumin; CHOL = cholesterol; TG = triglycerides; TP = total protein; CRE = creatinine; GLU = glucose; HDL-C = high-density lipoprotein cholesterol; LDL-C = low-density lipoprotein cholesterol.

**Table 6:** Effects of compound probiotics on serum immunity indexes of Holstein calves.

	Diet ^1^				
Item ^2^	CG (*n* = 10)	TG1 (*n* = 10)	TG2 (*n* = 10)	SEM	*p*-Value
IL-10 (pg/mL)	114.36	121.59	116.43	3.55	0.372
IL-6 (pg/mL)	400.73 ^a^	353.20 ^b^	412.44 ^a^	11.69	0.009
IL-2 (pg/mL)	242.99	254.15	253.81	11.65	0.749
TNF-α (pg/mL)	54.56	52.98	56.55	2.58	0.623
IFN-γ (pg/mL)	1062.03	1103.81	997.09	46.11	0.295
IgA (μg/mL)	1196.79	1213.00	1158.27	29.00	0.403
IgG (g/L)	12.17	11.55	11.16	0.49	0.356
IgM (μg/mL)	1819.06	1763.16	1850.02	79.30	0.749

^a,b^ Within a row, means without a common superscript differ (*p* < 0.05). ^1^ Diets were supplemented with different types of probiotic complexes: CG = control without probiotic complex; TG1 = basal diet + *Lactobacillus plantarum* + *Bacillus subtilis*; TG2 = basal diet + *Lactobacillus acidophilus* + *Bacillus subtilis*. Probiotic supplementation began at 3 days of age. ^2^ IL-10 = interleukin-10; IL-6 = interleukin-6; IL-2 = interleukin-2; TNF-α = tumor necrosis factor-α; IFN-γ = interferon-γ; IgA = immunoglobulin A; IgG = immunoglobulin G; IgM = immunoglobulin M.

**Table 7:** Effects of compound probiotics on rumen fermentation parameters of Holstein calves.

	Diet ^1^				
Item ^2^	CG (*n* = 10)	TG1 (*n* = 10)	TG2 (*n* = 10)	SEM	*p*-Value
pH	6.59	6.36	6.74	0.13	0.128
NH_3_-N (mg/100 mL)	3.37 ^a^	3.14 ^ab^	2.24 ^b^	0.32	0.026
BCP (mg/100 mL)	33.06 ^b^	44.07 ^a^	43.50 ^a^	2.24	0.009
TVFA (mmol/L)	64.07	78.90	57.74	8.06	0.407
Acetic acid (mmol/L)	30.85	40.30	34.19	4.77	0.435
Propionic acid (mmol/L)	21.52	23.88	14.57	2.42	0.176
Isobutyric acid (mmol/L)	1.12 ^b^	1.88 ^a^	1.01 ^ab^	0.23	0.030
Butyric acid (mmol/L)	5.01	6.16	4.15	1.35	0.110
Isovaleric acid (mmol/L)	2.13	2.76	1.88	0.38	0.341
Valeric acid (mmol/L)	3.44 ^ab^	3.92 ^a^	1.94 ^b^	0.59	0.045
Acetic: propionic	1.51	1.77	1.90	0.17	0.358

^a,b^ Within a row, means without a common superscript differ (*p* < 0.05). ^1^ Diets were supplemented with different types of probiotic complexes: CG = control without probiotic complex; TG1 = basal diet + *Lactobacillus plantarum* + *Bacillus subtilis*; TG2 = basal diet + *Lactobacillus acidophilus* + *Bacillus subtilis*. Probiotic supplementation began at 3 days of age. ^2^ NH_3_-N = ammonia nitrogen; BCP = bacterial crude protein; TVFA = total volatile fatty acids.

**Table 8:** Effects of compound probiotics on the Alpha diversity index of rumen bacteria of Holstein calves.

	Diet ^1^				
Item ^2^	CG (*n* = 10)	TG1 (*n* = 10)	TG2 (*n* = 10)	SEM	*p*-Value
ACE	178.48	165.07	164.47	0.74	0.491
Chao1	175.97	161.88	162.64	0.92	0.418
Simpson	0.12	0.14	0.12	0.08	0.872
Shannon	2.79	2.72	2.80	0.14	0.925

Within a row, means without a common superscript differ (*p* < 0.05). ^1^ Diets were supplemented with different types of probiotic complexes: CG = control without probiotic complex; TG1 = basal diet + *Lactobacillus plantarum* + *Bacillus subtilis*; TG2 = basal diet + *Lactobacillus acidophilus* + *Bacillus subtilis*. Probiotic supplementation began at 3 days of age. ^2^ ACE, Chao1, Simpson, and Shannon are Alpha diversity indices.

## Data Availability

The raw sequencing data of 16S rRNA sequences have been deposited in the NationalCenter for Biotechnology Information (NCBI) Sequence Read Archive (SRA) under BioProject accession number PRJNA1420454 https://dataview.ncbi.nlm.nih.gov/object/PRJNA1420454?reviewer=923ta9kvvideddpcckc1mm3nb1 (accessed on 1 December 2025), and the raw metabolomic data have been deposited in OMIX, China National Center for Bioinformation/Beijing Institute of Genomics, Chinese Academy of Sciences, under accession number OMIX015112 https://share.cncb.ac.cn/cKiwd5t8Yy/OMIX015112/ (accessed on 1 December 2025).

## Abbreviations

ADG, average daily gain; ALT, alanine aminotransferase; BCP, bacterial crude protein; CAT, catalase; CG, control group; CRE, creatinine; FCR, feed conversion ratio; GSH-Px, glutathione peroxidase; HDL-C, high-density lipoprotein cholesterol; NH_3_-N, ammonia nitrogen; OTU, operational taxonomic unit; VFA, volatile fatty acid.

## References

[1] Du W., Wang X., Hu M., Hou J., Du Y., Si W., Yang L., Xu L., Xu Q. (2023). Modulating Gastrointestinal Microbiota to Alleviate Diarrhea in Calves. Front. Microbiol..

[2] Dall Agnol A.M., Lorenzetti E., Leme R.A., Ladeia W.A., Mainardi R.M., Bernardi A., Headley S.A., Freire R.L., Pereira U.P., Alfieri A.F. (2021). Severe Outbreak of Bovine Neonatal Diarrhea in a Dairy Calf Rearing Unit with Multifactorial Etiology. Braz. J. Microbiol..

[3] Kim H.S., Whon T.W., Sung H., Jeong Y.S., Jung E.S., Shin N.R., Hyun D.W., Kim P.S., Lee J.Y., Lee C.H. (2021). Longitudinal Evaluation of Fecal Microbiota Transplantation for Ameliorating Calf Diarrhea and Improving Growth Performance. Nat. Commun..

[4] Soberon F., Van Amburgh M.E. (2013). Lactation Biology Symposium: The Effect of Nutrient Intake from Milk or Milk Replacer of Preweaned Dairy Calves on Lactation Milk Yield as Adults: A Meta-Analysis of Current Data. J. Anim. Sci..

[5] Urie N.J., Lombard J.E., Shivley C.B., Kopral C.A., Adams A.E., Earleywine T.J., Olson J.D., Garry F.B. (2018). Preweaned Heifer Management on Us Dairy Operations: Part, V. Factors Associated with Morbidity and Mortality in Preweaned Dairy Heifer Calves. J. Dairy Sci..

[6] Hill C., Guarner F., Reid G., Gibson G.R., Merenstein D.J., Pot B., Morelli L., Canani R.B., Flint H.J., Salminen S. (2014). Expert Consensus Document. The International Scientific Association for Probiotics and Prebiotics Consensus Statement on the Scope and Appropriate Use of the Term Probiotic. Nat. Rev. Gastroenterol. Hepatol..

[7] Gomez D.E., Arroyo L.G., Costa M.C., Viel L., Weese J.S. (2017). Characterization of the Fecal Bacterial Microbiota of Healthy and Diarrheic Dairy Calves. J. Vet. Intern. Med..

[8] Oikonomou G., Teixeira A.G., Foditsch C., Bicalho M.L., Machado V.S., Bicalho R.C. (2013). Fecal Microbial Diversity in Pre-Weaned Dairy Calves as Described by Pyrosequencing of Metagenomic 16S rDNA. Associations of Faecalibacterium Species with Health and Growth. PLoS ONE.

[9] Malmuthuge N., Liang G., Griebel P.J., Guan L.L. (2019). Taxonomic and Functional Compositions of the Small Intestinal Microbiome in Neonatal Calves Provide a Framework for Understanding Early Life Gut Health. Appl. Environ. Microbiol..

[10] Hu S., Wang L., Jiang Z. (2017). Dietary Additive Probiotics Modulation of the Intestinal Microbiota. Protein Pept. Lett..

[11] Stoeva M.K., Garcia-So J., Justice N., Myers J., Tyagi S., Nemchek M., McMurdie P.J., Kolterman O., Eid J. (2021). Butyrate-Producing Human Gut Symbiont, Clostridium Butyricum, and Its Role in Health and Disease. Gut Microbes.

[12] Casper D.P., Hultquist K.M., Acharya I.P. (2021). Lactobacillus Plantarum Gb Lp-1 as a Direct-fed Microbial for Neonatal Calves. J. Dairy Sci..

[13] Kumar M., Kala A., Chaudhary L.C., Agarwal N., Kochewad S.A. (2022). Microencapsulated and Lyophilized Lactobacillus Acidophilus Improved Gut Health and Immune Status of Preruminant Calves. Probiotics Antimicrob. Proteins.

[14] Antanaitis R., Džermeikaitė K., Krištolaitytė J., Armonavičiūtė E., Arlauskaitė S., Girdauskaitė A., Rutkauskas A., Baumgartner W. (2024). Effects of Bacillus Subtilis on Growth Performance, Metabolic Profile, and Health Status in Dairy Calves. Animals.

[15] Lucey P.M., Lean I.J., Aly S.S., Golder H.M., Block E., Thompson J.S., Rossow H.A. (2021). Effects of Mannan-Oligosaccharide and Bacillus Subtilis Supplementation to Preweaning Holstein Dairy Heifers on Body Weight Gain, Diarrhea, and Shedding of Fecal Pathogens. J. Dairy Sci..

[16] Wang Z., He Z., Beauchemin K.A., Tang S., Zhou C., Han X., Wang M., Kang J., Odongo N.E., Tan Z. (2016). Comparison of Two Live Bacillus Species as Feed Additives for Improving in Vitro Fermentation of Cereal Straws. Anim. Sci. J..

[17] Council, National Research (2001). Nutrient Requirements of Dairy Cattle: Seventh Revised Edition.

[18] Liu X., Sha Y., Dingkao R., Zhang W., Lv W., Wei H., Shi H., Hu J., Wang J., Li S. (2020). Interactions Between Rumen Microbes, Vfas, and Host Genes Regulate Nutrient Absorption and Epithelial Barrier Function During Cold Season Nutritional Stress in Tibetan Sheep. Front. Microbiol..

[19] Bradford M.M. (1976). A Rapid and Sensitive Method for the Quantitation of Microgram Quantities of Protein Utilizing the Principle of Protein-Dye Binding. Anal. Biochem..

[20] Magoč T., Salzberg S.L. (2011). Flash: Fast Length Adjustment of Short Reads to Improve Genome Assemblies. Bioinformatics.

[21] Edgar R.C. (2013). Uparse: Highly Accurate Otu Sequences from Microbial Amplicon Reads. Nat. Methods.

[22] Anderson C.J., Koester L.R., Schmitz-Esser S. (2021). Rumen Epithelial Communities Share a Core Bacterial Microbiota: A Meta-Analysis of 16S rRNA Gene Illumina Miseq Sequencing Datasets. Front. Microbiol..

[23] Chai J., Weiss C.P., Beck P.A., Zhao W., Li Y., Zhao J. (2024). Diet and Monensin Influence the Temporal Dynamics of the Rumen Microbiome in Stocker and Finishing Cattle. J. Anim. Sci. Biotechnol..

[24] Wang Q., Garrity G.M., Tiedje J.M., Cole J.R. (2007). Naive Bayesian Classifier for Rapid Assignment of rRNA Sequences into the New Bacterial Taxonomy. Appl. Environ. Microbiol..

[25] Razzaghi A., Valizadeh R., Naserian A.A., Mesgaran M.D., Carpenter A.J., Ghaffari M.H. (2016). Effect of Dietary Sugar Concentration and Sunflower Seed Supplementation on Lactation Performance, Ruminal Fermentation, Milk Fatty Acid Profile, and Blood Metabolites of Dairy Cows. J. Dairy Sci..

[26] Cai X., Yi P., Chen X., Wu J., Lan G., Li S., Luo S., Huang F., Huang J., Shen P. (2024). Intake of Compound Probiotics Accelerates the Construction of Immune Function and Gut Microbiome in Holstein Calves. Microbiol. Spectr..

[27] Zhang L., Liu X., Jia H. (2022). Wgcna Analysis of Important Modules and Hub Genes of Compound Probiotics Regulating Lipid Metabolism in Heat-Stressed Broilers. Animals.

[28] Wang H., Yu Z., Gao Z., Li Q., Qiu X., Wu F., Guan T., Cao B., Su H. (2022). Effects of Compound Probiotics on Growth Performance, Rumen Fermentation, Blood Parameters, and Health Status of Neonatal Holstein Calves. J. Dairy Sci..

[29] Wu Y., Nie C., Luo R., Qi F., Bai X., Chen H., Niu J., Chen C., Zhang W. (2021). Effects of Multispecies Probiotic on Intestinal Microbiota and Mucosal Barrier Function of Neonatal Calves Infected with, *E. coli* K99. Front. Microbiol..

[30] Frizzo L.S., Soto L.P., Zbrun M.V., Bertozzi E., Sequeira G., Armesto R.R., Rosmini M.R.J.A.F.S. (2010). Lactic Acid Bacteria to Improve Growth Performance in Young Calves Fed Milk Replacer and Spray-Dried Whey Powder. Anim. Feed. Sci. Technol..

[31] Hosseinian S.A., Hasanzadeh F. (2021). Impact of High Dietary Energy on Obesity and Oxidative Stress in Domestic Pigeons. Vet. Med. Sci..

[32] Qadis A.Q., Goya S., Yatsu M., Kimura A., Ichijo T., Sato S. (2014). Immune-Stimulatory Effects of a Bacteria-Based Probiotic on Peripheral Leukocyte Subpopulations and Cytokine Mrna Expression Levels in Scouring Holstein Calves. J. Vet. Med. Sci..

[33] Raabis S., Li W., Cersosimo L. (2019). Effects and Immune Responses of Probiotic Treatment in Ruminants. Vet. Immunol. Immunopathol..

[34] Ghorbani G.R., Morgavi D.P., Beauchemin K.A., Leedle J.A. (2002). Effects of Bacterial Direct-Fed Microbials on Ruminal Fermentation, Blood Variables, and the Microbial Populations of Feedlot Cattle. J. Anim. Sci..

[35] Jia P., Cui K., Ma T., Wan F., Wang W., Yang D., Wang Y., Guo B., Zhao L., Diao Q. (2018). Influence of Dietary Supplementation with *Bacillus licheniformis* and Saccharomyces Cerevisiae as Alternatives to Monensin on Growth Performance, Antioxidant, Immunity, Ruminal Fermentation and Microbial Diversity of Fattening Lambs. Sci. Rep..

[36] Guo Y.Q., Hu Y.R., Liu S.R., Wang M., Xian Z.Y., Liu D.W., Sun B.L., Li Y.K., Liu G.B., Deng M. (2023). Effects of the Oat Hay Feeding Method and Compound Probiotic Supplementation on the Growth, Antioxidant Capacity, Immunity, and Rumen Bacteria Community of Dairy Calves. Antioxid.

[37] Liu B., Wang C., Huasai S., Han A., Zhang J., He L., Aorigele C. (2022). Compound Probiotics Improve the Diarrhea Rate and Intestinal Microbiota of Newborn Calves. Animals.

[38] O’Hara E., Kenny D.A., McGovern E., Byrne C.J., McCabe M.S., Guan L.L., Waters S.M. (2020). Investigating Temporal Microbial Dynamics in the Rumen of Beef Calves Raised on Two Farms During Early Life. FEMS Microbiol. Ecol..

[39] Zhou J., Zhao K., Shao L., Bao Y., Gyantsen D., Ma C., Xue B. (2023). Effects of *Bacillus licheniformis* and Combination of Probiotics and Enzymes as Supplements on Growth Performance and Serum Parameters in Early-Weaned Grazing Yak Calves. Animals.

[40] Liang J., Tang S., Gong J., Zeng G., Tang W., Song B., Zhang P., Yang Z., Luo Y. (2020). Responses of Enzymatic Activity and Microbial Communities to Biochar/Compost Amendment in Sulfamethoxazole Polluted Wetland Soil. J. Hazard. Mater..

[41] Zhao C., Wang L., Ke S., Chen X., Kenéz Á., Xu W., Wang D., Zhang F., Li Y., Cui Z. (2022). Yak Rumen Microbiome Elevates Fiber Degradation Ability and Alters Rumen Fermentation Pattern to Increase Feed Efficiency. Anim. Nutr..

[42] Wang J., Chen J., Gao M., Ouyang Z., Li Y., Liu D., Zhu M., Sun H. (2025). Research Progress on the Mechanism of Action and Screening Methods of Probiotics for Lowering Blood Lipid Levels. Foods.

